# Supplementary material for: Multi‐dataset identification of innovative feature genes and molecular mechanisms in keratoconus
Source: J Cell Mol Med. 2024 Sep 19;28(18):e70079. doi: 10.1111/jcmm.70079 (PMC11412914; doi:10.1111/jcmm.70079)
Supplement: Supplementary file 2 — Material S2. [file JCMM-28-e70079-s002.zip › 7.GSEA analysis/6.SNORA74B/KEGG_FOCAL_ADHESION.html]

Details for gene set KEGG\_FOCAL\_ADHESION[GSEA]

|  || Dataset | SNORA74B.group.cls#h\_versus\_l |
| Phenotype | group.cls#h\_versus\_l |
| Upregulated in class | l |
| GeneSet | KEGG\_FOCAL\_ADHESION |
| Enrichment Score (ES) | -0.38551563 |
| Normalized Enrichment Score (NES) | -1.3441706 |
| Nominal p-value | 0.13627255 |
| FDR q-value | 0.3112607 |
| FWER p-Value | 0.931 |
Table: GSEA Results Summary

  

Fig 1: Enrichment plot: KEGG\_FOCAL\_ADHESION      
 Profile of the Running ES Score & Positions of GeneSet Members on the Rank Ordered List

  

| SYMBOL | TITLE | RANK IN GENE LIST | RANK METRIC SCORE | RUNNING ES | CORE ENRICHMENT || 1 | PIK3R5 | na | 170 | 0.800 | 0.0211 | No |
| 2 | FN1 | na | 587 | 0.575 | 0.0277 | No |
| 3 | COL1A1 | na | 1692 | 0.398 | 0.0087 | No |
| 4 | RAC3 | na | 1795 | 0.390 | 0.0184 | No |
| 5 | ELK1 | na | 2088 | 0.363 | 0.0218 | No |
| 6 | LAMB4 | na | 2399 | 0.339 | 0.0238 | No |
| 7 | PRKCG | na | 2539 | 0.330 | 0.0305 | No |
| 8 | PDGFRA | na | 4146 | 0.255 | -0.0077 | No |
| 9 | COL3A1 | na | 4518 | 0.242 | -0.0106 | No |
| 10 | COL6A6 | na | 5195 | 0.219 | -0.0231 | No |
| 11 | BCAR1 | na | 5506 | 0.208 | -0.0253 | No |
| 12 | COL1A2 | na | 5952 | 0.194 | -0.0319 | No |
| 13 | SOS1 | na | 5971 | 0.193 | -0.0261 | No |
| 14 | VWF | na | 6031 | 0.191 | -0.0216 | No |
| 15 | FLNB | na | 6092 | 0.190 | -0.0172 | No |
| 16 | IGF1R | na | 6399 | 0.180 | -0.0202 | No |
| 17 | MYL5 | na | 6916 | 0.165 | -0.0298 | No |
| 18 | COL4A1 | na | 7227 | 0.156 | -0.0337 | No |
| 19 | ITGA4 | na | 7435 | 0.150 | -0.0348 | No |
| 20 | ITGB7 | na | 7862 | 0.139 | -0.0426 | No |
| 21 | BRAF | na | 8089 | 0.134 | -0.0448 | No |
| 22 | PIK3R1 | na | 8116 | 0.133 | -0.0413 | No |
| 23 | IBSP | na | 8429 | 0.126 | -0.0462 | No |
| 24 | IGF1 | na | 8530 | 0.124 | -0.0451 | No |
| 25 | TNR | na | 8827 | 0.118 | -0.0498 | No |
| 26 | ROCK1 | na | 8851 | 0.117 | -0.0467 | No |
| 27 | LAMA5 | na | 9197 | 0.109 | -0.0531 | No |
| 28 | RASGRF1 | na | 9313 | 0.107 | -0.0530 | No |
| 29 | AKT2 | na | 9380 | 0.105 | -0.0515 | No |
| 30 | FLT4 | na | 9545 | 0.102 | -0.0529 | No |
| 31 | ITGA9 | na | 9643 | 0.100 | -0.0525 | No |
| 32 | ITGB6 | na | 9755 | 0.098 | -0.0525 | No |
| 33 | PDGFA | na | 9861 | 0.095 | -0.0525 | No |
| 34 | VAV2 | na | 10064 | 0.091 | -0.0554 | No |
| 35 | MAPK9 | na | 10427 | 0.085 | -0.0631 | No |
| 36 | TNXB | na | 10865 | 0.077 | -0.0732 | No |
| 37 | LAMC3 | na | 10898 | 0.077 | -0.0717 | No |
| 38 | PDPK1 | na | 11353 | 0.069 | -0.0826 | No |
| 39 | ACTB | na | 11421 | 0.068 | -0.0823 | No |
| 40 | ACTN4 | na | 12377 | 0.055 | -0.1082 | No |
| 41 | XIAP | na | 12442 | 0.054 | -0.1083 | No |
| 42 | SPP1 | na | 12450 | 0.054 | -0.1067 | No |
| 43 | PTEN | na | 12756 | 0.050 | -0.1139 | No |
| 44 | ERBB2 | na | 12767 | 0.050 | -0.1126 | No |
| 45 | VASP | na | 12788 | 0.049 | -0.1116 | No |
| 46 | PIK3R2 | na | 12946 | 0.047 | -0.1146 | No |
| 47 | PIK3CA | na | 13079 | 0.046 | -0.1169 | No |
| 48 | THBS3 | na | 13444 | 0.042 | -0.1261 | No |
| 49 | COL2A1 | na | 13535 | 0.041 | -0.1273 | No |
| 50 | SOS2 | na | 13685 | 0.039 | -0.1304 | No |
| 51 | PDGFRB | na | 14565 | 0.029 | -0.1549 | No |
| 52 | THBS1 | na | 14896 | 0.025 | -0.1636 | No |
| 53 | MAPK10 | na | 14996 | 0.024 | -0.1657 | No |
| 54 | CCND1 | na | 15223 | 0.022 | -0.1715 | No |
| 55 | FLT1 | na | 15419 | 0.020 | -0.1765 | No |
| 56 | DIAPH1 | na | 15556 | 0.019 | -0.1798 | No |
| 57 | PARVG | na | 15578 | 0.019 | -0.1798 | No |
| 58 | PIK3CB | na | 15769 | 0.017 | -0.1848 | No |
| 59 | MYL12B | na | 16040 | 0.014 | -0.1921 | No |
| 60 | VAV3 | na | 16097 | 0.014 | -0.1933 | No |
| 61 | ARHGAP35 | na | 16478 | 0.010 | -0.2040 | No |
| 62 | MAPK3 | na | 16753 | 0.007 | -0.2117 | No |
| 63 | BIRC2 | na | 16995 | 0.005 | -0.2185 | No |
| 64 | COL6A1 | na | 17070 | 0.004 | -0.2205 | No |
| 65 | PAK4 | na | 17175 | 0.003 | -0.2234 | No |
| 66 | PPP1CB | na | 17283 | 0.002 | -0.2265 | No |
| 67 | EGF | na | 17351 | 0.001 | -0.2284 | No |
| 68 | CAPN2 | na | 17361 | 0.001 | -0.2286 | No |
| 69 | ITGB4 | na | 17477 | -0.000 | -0.2319 | No |
| 70 | ROCK2 | na | 17655 | -0.002 | -0.2370 | No |
| 71 | DOCK1 | na | 17724 | -0.003 | -0.2389 | No |
| 72 | CTNNB1 | na | 17783 | -0.003 | -0.2404 | No |
| 73 | RAP1A | na | 17815 | -0.004 | -0.2412 | No |
| 74 | GSK3B | na | 17871 | -0.004 | -0.2427 | No |
| 75 | ACTG1 | na | 18268 | -0.009 | -0.2538 | No |
| 76 | RHOA | na | 18290 | -0.009 | -0.2541 | No |
| 77 | AKT1 | na | 18366 | -0.010 | -0.2560 | No |
| 78 | PPP1CA | na | 18424 | -0.011 | -0.2573 | No |
| 79 | PIP5K1C | na | 18480 | -0.012 | -0.2585 | No |
| 80 | MYL12A | na | 18525 | -0.012 | -0.2594 | No |
| 81 | RAP1B | na | 18593 | -0.013 | -0.2609 | No |
| 82 | VEGFB | na | 18662 | -0.013 | -0.2624 | No |
| 83 | CDC42 | na | 18770 | -0.015 | -0.2650 | No |
| 84 | COL6A3 | na | 18877 | -0.016 | -0.2676 | No |
| 85 | PIK3CG | na | 18884 | -0.016 | -0.2673 | No |
| 86 | RAC2 | na | 18892 | -0.016 | -0.2670 | No |
| 87 | PAK6 | na | 18984 | -0.017 | -0.2690 | No |
| 88 | LAMA4 | na | 19146 | -0.019 | -0.2731 | No |
| 89 | BAD | na | 19265 | -0.021 | -0.2758 | No |
| 90 | HGF | na | 19424 | -0.022 | -0.2797 | No |
| 91 | ARHGAP5 | na | 19489 | -0.023 | -0.2808 | No |
| 92 | EGFR | na | 19596 | -0.024 | -0.2831 | No |
| 93 | HRAS | na | 19699 | -0.025 | -0.2852 | No |
| 94 | RAC1 | na | 19881 | -0.027 | -0.2896 | No |
| 95 | COL11A2 | na | 20062 | -0.029 | -0.2939 | No |
| 96 | SHC3 | na | 20230 | -0.031 | -0.2977 | No |
| 97 | VTN | na | 20282 | -0.031 | -0.2982 | No |
| 98 | LAMB2 | na | 20349 | -0.032 | -0.2990 | No |
| 99 | PIK3CD | na | 20354 | -0.032 | -0.2981 | No |
| 100 | MAPK8 | na | 20414 | -0.033 | -0.2987 | No |
| 101 | MYLK | na | 20559 | -0.035 | -0.3018 | No |
| 102 | MAPK1 | na | 20606 | -0.035 | -0.3020 | No |
| 103 | COL5A3 | na | 20986 | -0.040 | -0.3116 | No |
| 104 | CCND2 | na | 21016 | -0.040 | -0.3112 | No |
| 105 | RAF1 | na | 21074 | -0.041 | -0.3115 | No |
| 106 | ILK | na | 21133 | -0.042 | -0.3118 | No |
| 107 | PAK3 | na | 21207 | -0.042 | -0.3126 | No |
| 108 | PPP1CC | na | 21514 | -0.046 | -0.3199 | No |
| 109 | ITGA2 | na | 22079 | -0.053 | -0.3346 | No |
| 110 | PRKCA | na | 22239 | -0.055 | -0.3374 | No |
| 111 | PPP1R12A | na | 22318 | -0.056 | -0.3378 | No |
| 112 | ITGA11 | na | 22351 | -0.056 | -0.3369 | No |
| 113 | VCL | na | 22408 | -0.057 | -0.3367 | No |
| 114 | COL11A1 | na | 22542 | -0.059 | -0.3387 | No |
| 115 | PTK2 | na | 22693 | -0.061 | -0.3410 | No |
| 116 | SHC1 | na | 22752 | -0.062 | -0.3407 | No |
| 117 | COL4A2 | na | 22912 | -0.064 | -0.3432 | No |
| 118 | ITGA3 | na | 23349 | -0.071 | -0.3536 | No |
| 119 | ZYX | na | 23765 | -0.077 | -0.3631 | No |
| 120 | ITGA2B | na | 23836 | -0.078 | -0.3625 | No |
| 121 | MYL2 | na | 23893 | -0.079 | -0.3616 | No |
| 122 | LAMB1 | na | 23959 | -0.080 | -0.3609 | No |
| 123 | LAMA2 | na | 24013 | -0.081 | -0.3598 | No |
| 124 | TLN2 | na | 24240 | -0.084 | -0.3636 | No |
| 125 | CHAD | na | 24249 | -0.085 | -0.3611 | No |
| 126 | ITGB5 | na | 24428 | -0.088 | -0.3634 | No |
| 127 | MYLK2 | na | 24506 | -0.089 | -0.3627 | No |
| 128 | BIRC3 | na | 24774 | -0.094 | -0.3673 | No |
| 129 | TNN | na | 24921 | -0.097 | -0.3684 | No |
| 130 | GRB2 | na | 24997 | -0.098 | -0.3674 | No |
| 131 | ITGA7 | na | 25192 | -0.102 | -0.3697 | No |
| 132 | VEGFA | na | 25195 | -0.102 | -0.3664 | No |
| 133 | CCND3 | na | 25274 | -0.103 | -0.3654 | No |
| 134 | JUN | na | 25317 | -0.104 | -0.3632 | No |
| 135 | TNC | na | 25442 | -0.106 | -0.3633 | No |
| 136 | PAK2 | na | 25754 | -0.112 | -0.3687 | No |
| 137 | PXN | na | 26060 | -0.118 | -0.3737 | No |
| 138 | LAMC2 | na | 26097 | -0.119 | -0.3709 | No |
| 139 | THBS4 | na | 26603 | -0.130 | -0.3813 | Yes |
| 140 | ITGAV | na | 26724 | -0.133 | -0.3804 | Yes |
| 141 | CRK | na | 26740 | -0.134 | -0.3765 | Yes |
| 142 | MYLK3 | na | 26822 | -0.135 | -0.3745 | Yes |
| 143 | TLN1 | na | 26949 | -0.138 | -0.3736 | Yes |
| 144 | LAMC1 | na | 27122 | -0.142 | -0.3740 | Yes |
| 145 | THBS2 | na | 27307 | -0.147 | -0.3745 | Yes |
| 146 | ITGB3 | na | 27418 | -0.149 | -0.3729 | Yes |
| 147 | ITGB1 | na | 27543 | -0.152 | -0.3715 | Yes |
| 148 | RELN | na | 27685 | -0.156 | -0.3705 | Yes |
| 149 | LAMA3 | na | 27891 | -0.162 | -0.3712 | Yes |
| 150 | ITGA6 | na | 27950 | -0.163 | -0.3676 | Yes |
| 151 | ACTN1 | na | 28221 | -0.171 | -0.3698 | Yes |
| 152 | PAK1 | na | 28478 | -0.179 | -0.3714 | Yes |
| 153 | FLNC | na | 28751 | -0.188 | -0.3732 | Yes |
| 154 | MET | na | 28809 | -0.189 | -0.3687 | Yes |
| 155 | RAPGEF1 | na | 28816 | -0.189 | -0.3627 | Yes |
| 156 | COL5A1 | na | 28983 | -0.194 | -0.3612 | Yes |
| 157 | LAMB3 | na | 29125 | -0.198 | -0.3589 | Yes |
| 158 | CRKL | na | 29271 | -0.203 | -0.3565 | Yes |
| 159 | PIK3R3 | na | 29453 | -0.208 | -0.3549 | Yes |
| 160 | ITGA10 | na | 29479 | -0.209 | -0.3489 | Yes |
| 161 | COL4A4 | na | 30027 | -0.227 | -0.3574 | Yes |
| 162 | PDGFB | na | 30041 | -0.227 | -0.3504 | Yes |
| 163 | FLNA | na | 30062 | -0.228 | -0.3435 | Yes |
| 164 | PGF | na | 30172 | -0.232 | -0.3391 | Yes |
| 165 | SRC | na | 30227 | -0.234 | -0.3331 | Yes |
| 166 | ACTN2 | na | 30345 | -0.239 | -0.3287 | Yes |
| 167 | MYL9 | na | 30587 | -0.249 | -0.3276 | Yes |
| 168 | PARVA | na | 30696 | -0.253 | -0.3225 | Yes |
| 169 | MAP2K1 | na | 31129 | -0.275 | -0.3261 | Yes |
| 170 | COL5A2 | na | 31293 | -0.283 | -0.3216 | Yes |
| 171 | ITGB8 | na | 32130 | -0.330 | -0.3351 | Yes |
| 172 | COMP | na | 32169 | -0.332 | -0.3255 | Yes |
| 173 | KDR | na | 32219 | -0.336 | -0.3160 | Yes |
| 174 | SHC4 | na | 32485 | -0.353 | -0.3122 | Yes |
| 175 | VAV1 | na | 32928 | -0.388 | -0.3124 | Yes |
| 176 | COL4A6 | na | 33172 | -0.411 | -0.3061 | Yes |
| 177 | ITGA5 | na | 33190 | -0.413 | -0.2931 | Yes |
| 178 | ACTN3 | na | 33200 | -0.413 | -0.2800 | Yes |
| 179 | PARVB | na | 33219 | -0.415 | -0.2670 | Yes |
| 180 | MYL7 | na | 33280 | -0.422 | -0.2550 | Yes |
| 181 | COL6A2 | na | 33508 | -0.450 | -0.2470 | Yes |
| 182 | CAV1 | na | 33529 | -0.453 | -0.2328 | Yes |
| 183 | FYN | na | 33540 | -0.454 | -0.2184 | Yes |
| 184 | ITGA8 | na | 33651 | -0.470 | -0.2063 | Yes |
| 185 | LAMA1 | na | 33685 | -0.475 | -0.1918 | Yes |
| 186 | BCL2 | na | 33746 | -0.485 | -0.1778 | Yes |
| 187 | CAV2 | na | 33835 | -0.501 | -0.1640 | Yes |
| 188 | ITGA1 | na | 33862 | -0.506 | -0.1483 | Yes |
| 189 | PRKCB | na | 33882 | -0.508 | -0.1324 | Yes |
| 190 | MYL10 | na | 34073 | -0.549 | -0.1200 | Yes |
| 191 | VEGFC | na | 34267 | -0.599 | -0.1062 | Yes |
| 192 | PDGFC | na | 34382 | -0.645 | -0.0885 | Yes |
| 193 | SHC2 | na | 34407 | -0.652 | -0.0680 | Yes |
| 194 | PDGFD | na | 34541 | -0.723 | -0.0484 | Yes |
| 195 | CAV3 | na | 34584 | -0.774 | -0.0244 | Yes |
| 196 | AKT3 | na | 34657 | -0.880 | 0.0021 | Yes |
Table: GSEA details [plain text format]

  

Fig 2: KEGG\_FOCAL\_ADHESION      
 Blue-Pink O' Gram in the Space of the Analyzed GeneSet

  

Fig 3: KEGG\_FOCAL\_ADHESION: Random ES distribution      
 Gene set null distribution of ES for **KEGG\_FOCAL\_ADHESION**
